# Collateral anastomosis in Leriche syndrome complicated by presumed mycotic thoracoabdominal aneurysm

**DOI:** 10.1016/j.radcr.2025.12.054

**Published:** 2026-01-21

**Authors:** Alexandros Apostolou, Maria Kadditi, Ilias G. Koziakas, Abdallah Aburub, Matthias Eberhard

**Affiliations:** a Department of Diagnostic and Interventional Radiology, Zurich University Hospital (Universitätsspital Zürich), Rämistrasse 100, 8091, Zurich, Switzerland; b Department of Radiology, Penteli Children’s Hospital, Ippokratous 8, Penteli 15236, Athens, Greece; c Department of Anesthesiology, Onassio Cardiac Surgery Center, Leof. Andrea Siggrou 356, Kallithea 17674, Athens, Greece; d Department of Diagnostic and Interventional Radiology, Marburg University Hospital (Uniklinikum Marburg UKGM), Baldingerstrasse 1, 35043, Marburg, Germany

**Keywords:** Leriche syndrome, Collaterals, Mycotic aneurysm, Thrombophilia, Intravenous drug

## Abstract

Leriche syndrome can remain clinically silent when collateral pathways preserve lower-limb perfusion; however, coexistence with infectious aortic pathology poses a considerable diagnostic challenge for the radiologist. We report a rare case of a 49-year-old male who presented with acute back pain and dysphagia; CT imaging demonstrated a chronic aortoiliac occlusion alongside a suspected mycotic thoracoabdominal aortic aneurysm. Notably, perfusion of the lower limbs was entirely dependent on bilateral internal thoracic–epigastric collateral pathways. This unusual combination highlights the broad spectrum of vascular complications associated with intravenous drug abuse and thrombophilia.

## Introduction

Leriche syndrome refers to chronic aortoiliac occlusive disease caused by progressive obstruction of the infrarenal aorta and/or iliac arteries, most commonly due to advanced atherosclerosis. It predominantly affects middle-aged to older individuals with cardiovascular risk factors, though earlier presentation may occur in the presence of thrombophilia or other prothrombotic conditions. Gradual disease progression allows the development of extensive collateral networks, allowing partial or complete preservation of lower-limb perfusion and leading to delayed, atypical, or even absent ischemic symptoms [1]. Mycotic aortic aneurysms are rare, accounting for approximately 0.7%-3% of all aortic aneurysms, but are associated with disproportionately high morbidity and mortality. They arise from infectious involvement of the arterial wall, leading to rapid mural destruction and aneurysmal degeneration. Predisposing factors include immunosuppression, intravenous drug use, diabetes mellitus, and pre-existing atherosclerotic disease. The clinical presentation is often nonspecific, with symptoms such as pain, fever, or systemic inflammation, which may delay diagnosis [2].

## Case report

A 49-year-old male with a history of chronic polytoxico- mania—including daily nicotine and cannabis use as well as long-term heroin consumption—presented with acute lower back pain for 2 days, dysphagia for 5 days, and unintentional weight loss of approximately 2-3 kg. He denied fever, night sweats, or other B-symptoms. His medical history included a known heterozygous prothrombin G20210A mutation.

Upon physical examination, the patient appeared cachectic but hemodynamically stable. Peripheral vascular assessment revealed weak but palpable bilateral femoral pulses, cool lower extremities and prolonged capillary refill. Neurological examination showed preserved motor strength and intact sensation in both legs; no signs of acute limb-threatening ischemia were present. Routine blood tests demonstrated mildly elevated inflammatory markers.

**Fig. 1:**
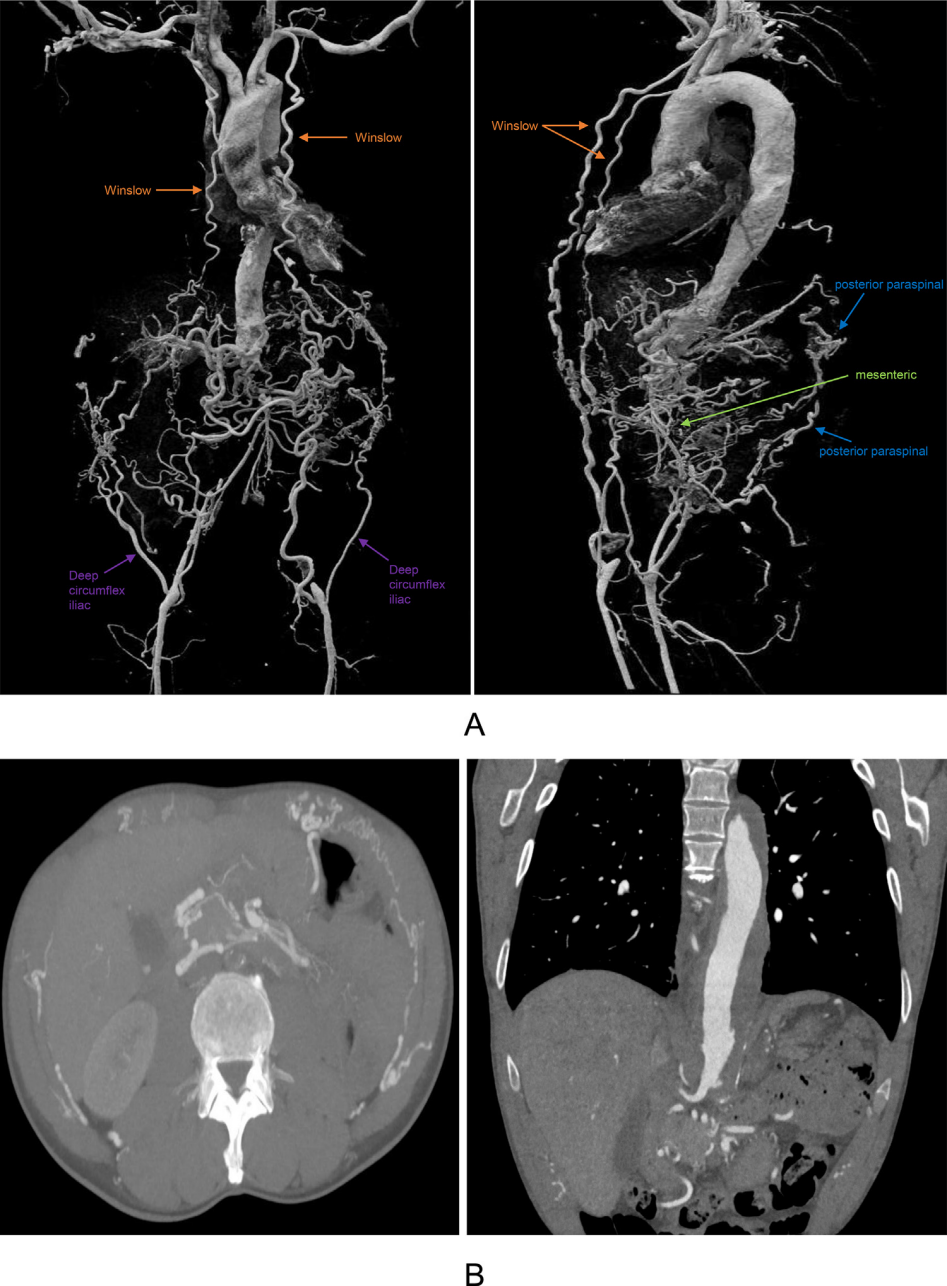
(A) Cinematic VRT demonstrating aortoiliac occlusion with bilateral Winslow pathway and additional collateral circuits (posterior paraspinal, mesenteric, deep circumflex iliac) and (B) Multiplanar CTA demonstrating aortoiliac occlusion with bilateral Winslow pathway and additional collateral circuits (posterior paraspinal, mesenteric, deep circumflex iliac).

**Fig. 2:**
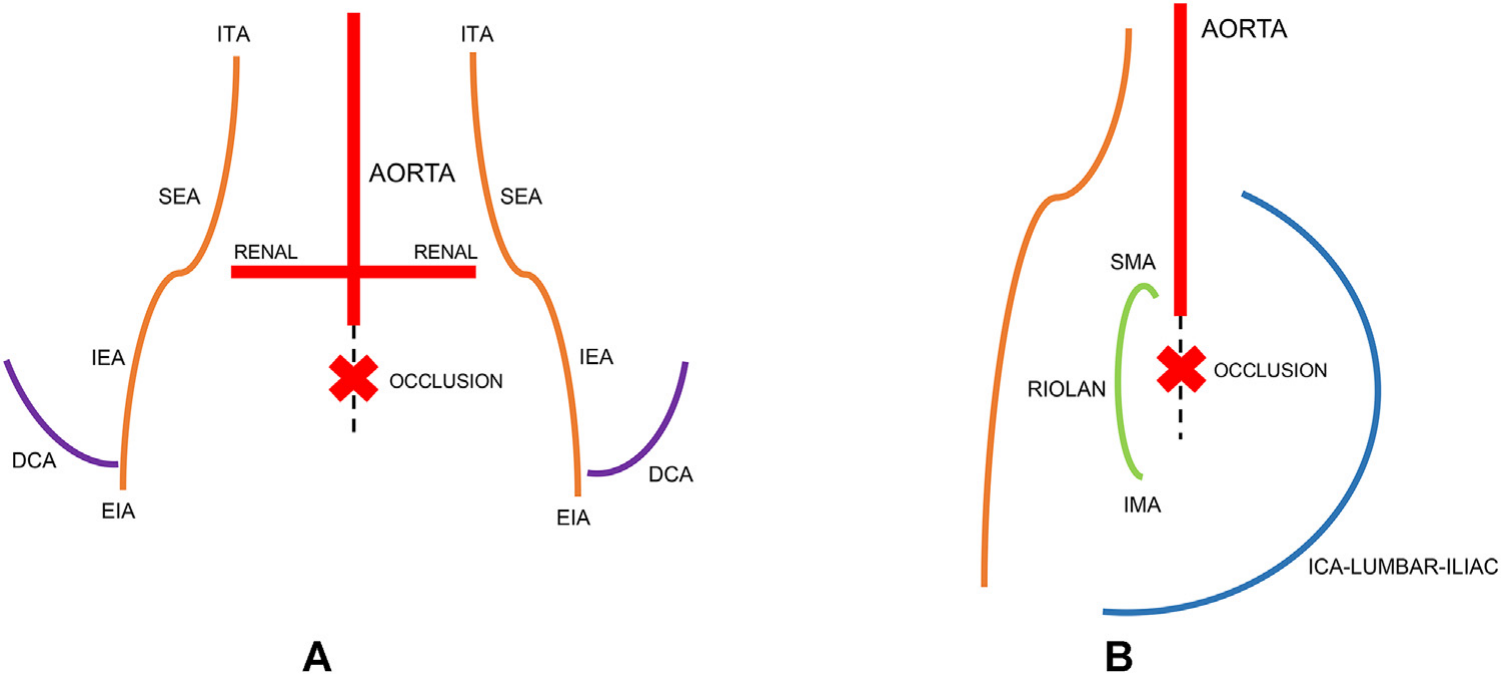
(A) Bilateral Internal Thoracic–Epigastric (Winslow) Pathway with schematic anterior view highlighting bilateral ITA→SEA→IEA→EIA collateralization with infrarenal aortic occlusion marked below the renal arteries and (B) Alternative Collateral Pathways with schematic lateral view showing posterior intercostal→lumbar→iliac collaterals, SMA↔IMA via the arc of Riolan/marginal artery and deep circumflex iliac pathways; infrarenal aortic occlusion marked below the renal arteries. (DCA, deep circumflex iliac artery; EIA, external iliac artery; ITA, internal thoracic artery; IMA, inferior mesenteric artery; ICA, intercostal artery; IEA, inferior epigastric artery; SEA, superior epigastric artery; SMA, superior mesenteric artery).

Work-up with computed tomography angiography (CTA) of the thorax and abdomen yielded a chronic complete occlusion of the infrarenal aorta, consistent with Leriche syndrome (Figs. 1 and 2). There was prominent collateralization, most strikingly via a bilateral internal thoracic–superior epigastric–inferior epigastric (Winslow) pathway, with hypertrophied internal thoracic arteries continuing as superior epigastric arteries and anastomosing with enlarged inferior epigastric arteries to reconstitute the right proximal and left distal external iliac artery. In addition to the dominant anterior Winslow axis, secondary collateral circuits were also appreciable, including posterior intercostal–lumbar–iliac pathways, mesenteric collateralization via the superior mesenteric artery to inferior mesenteric artery connections (arc of Riolan/marginal artery), as well as deep circumflex iliac pathways.

**Fig. 3:**
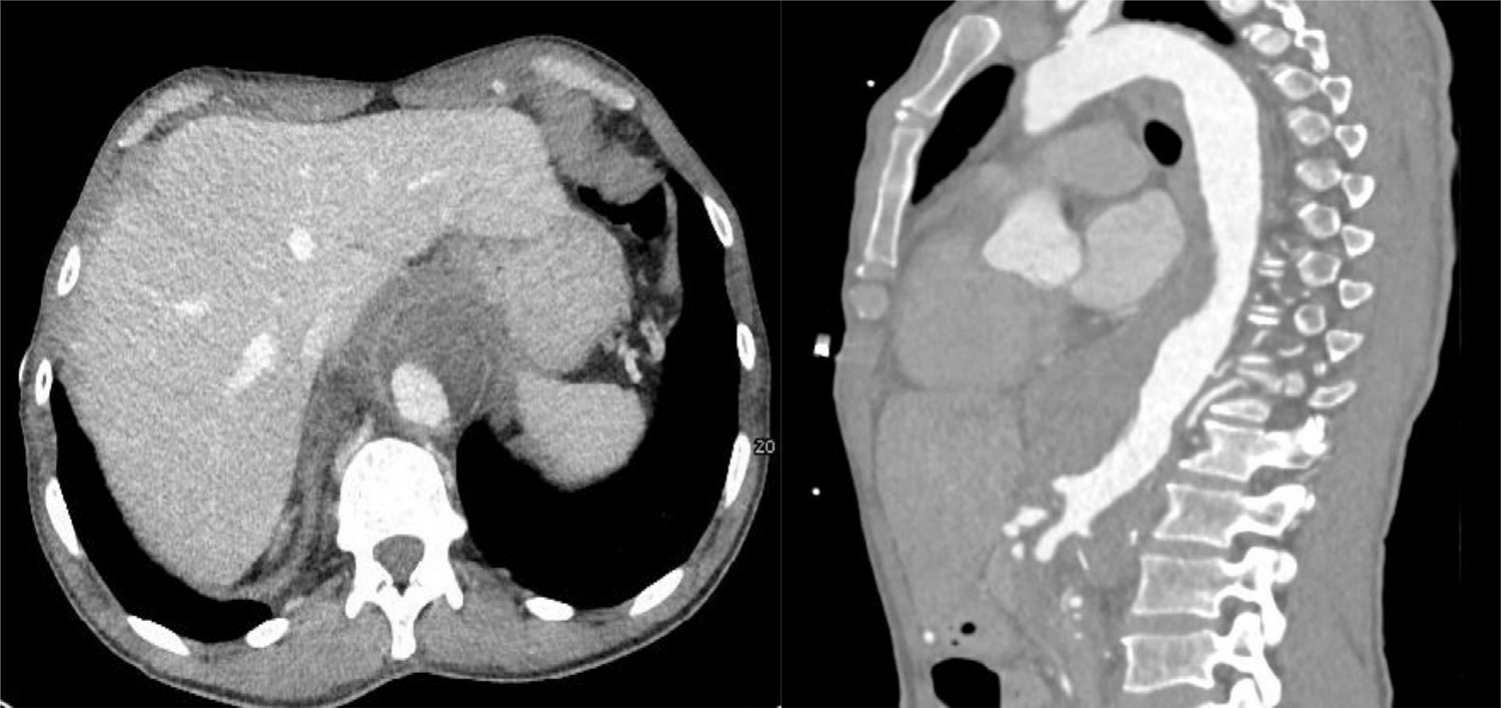
CTA showing lobulated thoracoabdominal aneurysm with periaortic inflammatory changes, soft-tissue cuff, and lymph node manchette, consistent with suspected mycotic aneurysm.

A second clinically relevant finding was a lobulated thoracoabdominal aortic aneurysm (TAAA) displaying imaging characteristics highly suggestive of a mycotic (infected) aneurysm on CTA according to recent radiologic and vascular surgery literature (Fig. 3) [3,4]. The aneurysm demonstrated periaortic fat stranding, soft-tissue inflammatory cuffing, periaortic lymphadenopathy (“lymph node manchette”), and irregular arterial wall morphology, all of which are recognized CT markers of infection-related aneurysmal disease. No evidence of acute rupture, whether contained or free.

Given the presence of cool lower extremities with palpable peripheral pulses and preserved distal perfusion, an initial conservative management strategy was pursued. Antithrombotic therapy with acetylsalicylic acid and therapeutic anticoagulation with heparin was initiated given the known prothrombin G20210A mutation. As the thoracoabdominal aortic aneurysm measured approximately 5 cm in maximum diameter and showed no morphologic features mandating urgent intervention, surgical repair was deferred. In the absence of fever, clinical signs of systemic infection or markedly elevated inflammatory parameters, antibiotic therapy was not initiated. A comprehensive coagulation and thrombophilia assessment was planned prior to transition to oral anticoagulation. Close radiologic surveillance with short-interval follow-up computed tomography was implemented, with surgical intervention reserved for the development of critical limb ischemia or aneurysmal progression. The patient was discharged home in stable clinical condition with structured outpatient follow-up.

## Discussion

This case highlights the remarkable adaptive capacity of the anterior thoracoabdominal wall collateral circulation in chronic aortoiliac occlusion. In complete infrarenal aortic obstruction, the internal thoracic–epigastric (Winslow) pathway can evolve into the dominant collateral pathway to the lower extremities. In this pathway, the internal thoracic arteries continue as the superior epigastric arteries, which then anastomose with hypertrophied inferior epigastric arteries that reconstitute the external iliac vessels. This collateral system has been described in classic angiographic and surgical literature as a key compensatory circuit in Leriche syndrome and other proximal inflow lesions, and modern imaging has reaffirmed its relevance with contemporary CTA and MRA techniques [[5], [6], [7]], but also as a potential source of catastrophic ischemia if disrupted. Its clinical importance is underscored by reports of acute lower-limb ischemia following internal thoracic artery harvest for coronary artery bypass graft surgery when this vessel constituted the principal collateral inflow to the legs [8,9].

The relatively young age of this patient for such advanced aortoiliac occlusive disease warrants consideration of contributing factors that likely accelerated vascular pathology. Chronic nicotine use promotes oxidative stress, endothelial dysfunction, and atherogenesis, thereby hastening peripheral arterial disease [10]. Cannabis consumption has increasingly been linked to vascular inflammation, arteritis, and vasospasm [11]. Heroin and intravenous/intra-arterial drug use (IDU) further compound vascular risk by promoting endothelial injury, vasospasm, particulate embolization, and recurrent bacteremia [12]. In addition, the patient’s heterozygous prothrombin G20210A mutation increases circulating prothrombin levels and is recognized as a significant heritable thrombophilia, with greater clinical impact in younger individuals and in the presence of acquired prothrombotic exposures [13,14]. The combination of these risk factors very likely contributed to premature development of aortoiliac occlusion in this case.

The CTA also revealed a lobulated thoracoabdominal aortic aneurysm (TAAA) with periaortic inflammatory tissue and lymphadenopathy, raising concern for a mycotic (infected) aneurysm. International literature emphasizes that people who inject drugs (PWID) are at disproportionally high risk of infected aneurysms, most frequently involving the lower limb, due to recurrent bacteremia, direct vessel injury, and contamination of injected substances [15]. Aortic involvement occur secondary through hematogenous seeding resulting in suppuration, mural destruction, and subsequent perforation or pseudoaneurysm formation. [16]. Contemporary data highlight the aggressive natural history of mycotic aneurysms, including rapid expansion, risk of rupture, and potential formation of aortoenteric or aortoesophageal fistulas [17,18]; in thoracic involvement, mass effect or inflammatory adherence to the esophagus can manifest as dysphagia aortica, which corresponds well to this patient’s presenting symptoms [19].

Despite the striking imaging findings, the multidisciplinary team adopted an initial conservative management strategy in accordance with current clinical guidelines and best practice recommendations [[20], [21], [22]]. The decision reflected the absence of hemodynamic instability, critical limb ischemia, or morphologic features necessitating urgent surgical intervention, as well as the preserved distal perfusion and subthreshold aneurysmal diameter. This approach emphasized close clinical and radiologic surveillance while reserving invasive treatment for clearly defined progression or complication.

The coexistence of chronic aortoiliac occlusion and a presumed mycotic thoracoabdominal aneurysm in a single patient is highly unusual and expands the spectrum of vascular complications associated with IDU and thrombophilia. This combination underscores the need for heightened clinical awareness in similar high-risk patients and adds a novel contribution to the existing literature on Leriche syndrome and mycotic aortic pathology.

## Patient consent

Complete written informed consent was obtained from the patient for the publication of this study and accompanying images.
